# The Chimeric Self: A Neo Naturalist Bundle Theory of the Self

**DOI:** 10.3389/fpsyg.2019.00202

**Published:** 2019-02-07

**Authors:** Lucrezia Compiani

**Affiliations:** Department of Cognitive Sciences, Psychology, Education and Cultural Studies, Messina University, Messina, Italy

**Keywords:** Self, Neo Naturalism, identity, relative existence, first person experience, ontology, bundle theory, Spread Mind

## Abstract

In the contemporary cognitive science and philosophy of mind debate the definition of the ontology of the Self is difficult if not downright dubious. Thus, different theories aim to provide an account, especially where further neuroscientific research could be implemented. To this extent, the identity of the Self is suggested to be pinpointed by virtue of its specific set of mechanical features or brain functions, or it is considered the product of cognitive and conceptual capacities that build representations and narratives about ourselves. In this paper, I propose an alternative approach, based on the Spread Mind Theory (Manzotti, 2017a). Starting from the idea that the Self isn’t just an aprioristic or transcendental form, I claim, endorsing a Neo Naturalist approach, that our first-personal experience is identical to the external objects which, due to a physical relation, constitute the same experience. Thanks to an externalist explanation of the experience of the Self it is possible to avoid multiple ontologies, causal foundationalism, naïve materialism and questions begging about what we should explain. Clarifying the concept of the Self, as a bundle of relative objects which are identical with the experiences themselves, allows us to draw a physical ontology, based on the neutral (and natural) idea of relative existence rather than any posited concept of subjectivity.

## The Argument in Brief

Who are we? What are our first personal experiences? To what are we identical to? These questions set up my research.

The problem of the Self had always entered philosophical and scientific debate. In the last 50 years, the philosophy of mind and cognitive neuroscience have been based on a naturalistic framework to explain life and experience. So, in the contemporary debate many theories have competed: from constructivism, which considers subjectivity as a phenomenal product of complex cerebral functions (McKay, 1978, 1980; Vogeley and Fink, 2003; Damasio, 2010; Vogeley and Gallagher, 2011; Panksepp and Biven, 2012; Churchland, 2013), and phenomenology (Thompson, 2007, 2015; Zahavi, 2014), which argues that the subject itself is the hallmark of all experiences, namely the paramount condition to have experiences, whatever they may be; to eliminativism (Dennett, 1992; Metzinger, 2003), which, instead, claims that the phenomenal Self is merely an illusion created by our brain functioning. All of these views, as I will argue, share the same premises which lead those approaches to misunderstand the physical nature of the Self as a bundle of physical reality.

Starting from the abovementioned approaches, I endorse a different view, which I call Neo Naturalist Bundle Theory of the Self^1^. My point is straightforward: I claim that the concept of identity bundle is grounded on the (physical) fact that we are identical with our experiences. The identity between our experiences and ourselves underpins the identity bundle itself and its purpose. This theory, following Manzotti’s Spread Mind^2^ hypothesis (Manzotti, 2017a,b), claims that what is identical with our experiences are the objects that constitute those experiences. These objects are not our bodies, or a part of it, like the brain, but the object we can experience, and our experience is made of this (unless we are talking about an experience in which a part of our body can be the object of the experience itself). By relying on the premises set by SMT, I claim that it is possible to describe the identity bundle like the whole stream of objects experienced by an organism, because they are identical with the experience of that organism.

Neo Naturalist Bundle Theory of the Self is a realist and physicalist theory of the Self which uses only identity to explain what phenomenal experience is, without postulating non-physical proprieties as phenomenal properties or *qualia* (Ayer, 1940; Nagel, 1974; Shoemaker, 1994; Chalmers, 1996). The explanatory strategy adopted, which we could define Neo-Naturalist in its approach, endeavors to physically respond to the problem of subjective experience, proposing a physical correlation of experience which is just identical to it: the relative object which constitutes the experience itself. For example, let us imagine that we are seeing a tree in front of us, we are having a first personal experience of the tree; according to the SMT, our first-person experience is precisely identical with the physical object in front of us: the tree.

The paper is organized as follows: in the first part, I describe the empirical–phenomenal models of the Self. Analyzing the arguments and the premises behind them, I highlight how they rely on arbitrary assumptions, not empirically grounded but derived from conceptual premises inherited over time.

In the second part, I present an alternative proposal: the NNBTS. I claim that what we call the Self is simply the continuous stream of experiences that we live in. Starting from this claim, Neo-Naturalism endorses the idea that our experiences are not something inner to the body, like private, or subjective, mental representations, rather they are the bundle itself, they are world, namely the world made of relative objects actualized by our conditions of existence, drawn by the physiology of our bodies. The Self is real, it exists, but it is not the metaphysical, mathematical, or psychological structure that we hope to fix once for all, neither the phenomenal idea that we create about ourselves. The bundle of identity is simply the hectic stream of perceptions, or experiences, that we experience according to our physiological condition of existence; physically speaking, it is a hectic stream of relative objects.

In the last part, I try to test the NNBTS with a set of issues as: the alleged concept of body ownership based on the assumption that “we are our body”; and the concept of person which is grounded on the idea of permanence through time.

## Empirical–Phenomenal Models of the Self

From Plato, many scholars reflected upon the nature of the Self, but a crucial time was the 17th century, when Descartes and Galileo started to speak about science and subject in a modern way. Descartes’s conclusions are famous: the Self, thanks to its core capacity to make *clear and distinct ideas*, is the bedrock of the possibility of knowing the world in a correct way (today we would say in a scientific way). The subject is the thought and the thought is the guarantee for the subject’s existence: *Cogito ergo sum* (Descartes, 1641).

There are two main ideas in Cartesian philosophy that are key to understanding the Self. On the one hand, the mental nature of the Self, namely the fact that what is referred to by the word “Self” is identified with the mental activity of the subject itself; on the other hand, the marked separation between the physical ground of the subject, namely the body, and its true mental essence, the soul. In Descartes’s opinion, the Self is not constituted by the body, but it is a stand-alone entity, existing in its own ontological domain. The substance dualism (physical vs. mental) is the troublesome legacy Descartes left us.

Descartes’s dualism is justified by another modern dualism: that of Galileo. Galileo, in *The Assayer*, distinguished two ontologies, two classes of properties of the thing: the quantitative properties and the qualitative ones ^3^, becoming the first supporter of the ontological dualism of real (physical, more like mathematical) vs. mental (phenomenal, subjective, mental). In the Galilean model, the core qualities are the quantitative ones, namely shapes, movement and quantity, because they constitute the absolute reality of the thing. They are accessible and reachable only through the rational investigation of the mind that, thanks to the framework provided by mathematics, can understand them. The second group of properties, the phenomenal ones, are considered as mere appearances and therefore are epistemologically downgraded because they are based on fallacious sensations rather than on mathematical truths. They are considered an addition to the physical world, they are second attributes of reality since they are the mental qualities evoked by sensory perception.

During the 17th century significant progress was made in terms of mind, experience, reality, and subjectivity. The ideas put forward in this era have echoed until today and are still key. The contemporary debate about the Self starts in fact from Descartes’s doubts and, following Galileo’s legacy, tries to give a physicalist answer to the question: what are we? What is our place in the physical world? What is the experience of ourselves identical to?

Nowadays, the core purpose in the philosophical discussion is, in fact, no longer only focused on issues like “self-knowledge” or “immunity to error through misidentification relative to the first person,” as prescribed by analytic Wittgensteinian philosophy (Strawson, 1959; Shoemaker, 1968, 1994; Cassam, 1994, 1997; Perry, 2001, 2002); rather, nowadays, the goal is to naturalize subjective experience, phenomenal experience. It means to describe experience using the theoretical frameworks of natural sciences such as physics, biology, and neuroscience. Analytical philosophy itself tended toward a materialistic turn in philosophy of mind at first to understand the real nature of the human mind. In this sense, the statement of the American epistemologist Wilfrid Sellars is emblematic, who assertively claims that science is the measure of all things when we want to describe and explain what is real and what is not (Sellars, 1962).

Nevertheless, after 60 years of research on consciousness, analytic philosophers and neuroscientists still have to grapple with the hard problem of consciousness (Chalmers, 1996). Cognitive neuroscience has tried to naturalize phenomenal experience^4^ through some candidates: from cognitive functions to neurons, to properties that emerge from the relations between complex systems as brain, body, and environment (Armstrong, 1968; McKay, 1978, 1980; Crick, 1994; Zeki and Bartels, 1999; O’Regan and Noë, 2001; Noë, 2004; Thompson, 2007; Tononi, 2008; Tononi and Koch, 2008, 2014; Tononi and Laureys, 2009; O’Regan, 2011). To put it another way: neuroscientists, and cognitive scientists outline the first-personal experience of seeing an oak, for example, as identical with the activation of certain brain areas involved in a specific function (sight in this case). These brain activations produce a mental representation of the object standing in front of us, and give us something that we call the phenomenal experience of the oak.

However, the identity between experience (of seeing a tree, for example) and the brain is inadmissible, because any of the physical correlates proposed by cognitive scientists has the same features of first personal experience. The identity between mind and brain results untenable if it is not enriched by the Galilean creative solution of postulating an extra ontology, the phenomenal one, to explain the peculiarity of first-personal experiences (Manzotti, 2017a,b). Despite this undeniable explanatory gap, neurocognitive science supports the idea that there is identity between experiences and brain (Smart, 1959; Armstrong, 1968; Marr, 1982; Lewis, 1986; Crick, 1994; Zeki and Bartels, 1999; Koch, 2004; Tononi, 2004; Tononi and Laureys, 2009; Dehaene, 2014), or between the mind and the brain^5^. Conscious experience is described by cognitive neuroscientists through functional analysis, namely the articulated breakdown of the paramount mental functions, including the Self, into more specialized functions. Mental functions are then localized inside the anatomical structures of the brain, by marking the correlations between cerebral substrate and function (following the inheritance of phrenology) through various neuroscientific methods. By taking this approach, the Self is considered the activation of certain brain networks (Churchland, 2013) which generate something non-physical, namely a first-person character of experiences: the phenomenal experience itself.

Let us take a practical example of the mainstream approach to experience, even of first-personal experience. For example, I have an oak in front of me. My visual experience of the oak is, for cognitive neuroscience, identical with the computation of sensory data coming from the external world to my retina and elaborated by my visual cortex. The results of this computation (which can be both cognitive or sensory-motor) are the mental representations of the external input, these representations giving rise to the phenomenal, subjective, qualitative experience of the oak. Representations generate my phenomenal experience of the oak and in this way, they are like a link between physical phenomena. In some sense, representations have the same role of Descartes’ pineal gland: they are an *ad hoc* space for an impossible meeting between incompatible ontologies, as physical reality and phenomenal experiences are (Manzotti, 2017a).

Unfortunately, the identity between experience and brain is not empirically justified, so neuroscientists need to introduce the concept of representations. They claim that neural systems have the power to create phenomenal representations of the external world. These representations should be the qualitative properties of experience, the same properties that Galileo appointed as secondary and negligible in respect of the primary qualities of reality. However, representations do not have a physical nature. They are only useful heuristic tools with which we can describe the alleged phenomenal character of experience, but this usage is not enough to transform them in to real physical experiences (Manzotti, 2017a). Therefore, the naturalization of experience assumed the explanation of conscious experience within the naturalistic framework, but this intent has been disregarded.

Taking this materialistic description to be true, we are jumping outside the physical world, i.e., nature. It could amount to an institutionalized jump, but it is still a jump. The idea that the physical substrate (or the functional substrate) causes the mind, which presents a non-physical ontology, such as that of representations, has no clear meaning. It remains unclear how a physical thing can generate non-physical things, such as representations; furthermore, it is unclear how a thing can be non-physical. We can perceive, and know, only physical things.

From this approach over the last 30 years, the empirical–phenomenal models of the Self have emerged, namely all those philosophical-scientific perspectives that intend to reduce the Self to a mere phenomenal product of the complex physical functioning of the brain (McKay, 1978, 1980; Dennett, 1988, 1992; Devinsky, 2000; Kircher et al., 2000; Metzinger, 2003; Platek et al., 2003; Northoff et al., 2006; Tononi and Laureys, 2009; Panksepp and Biven, 2012; Churchland, 2013). Following the atomistic rules laid down by the method of functional analysis, nowadays the Self is described as a set of different phenomenal experiences which characterize the Self as agent (Farrer and Frith, 2002; Tsakiris and Haggard, 2005; Gallese and Sinigaglia, 2010, 2011), as storyteller (Bruner, 1987; Dennett, 1992), as social Self (Vogeley et al., 2001; Gergen, 2011), as embodied Self (Gallese and Ferri, 2014) etc. The explanatory strategy adopted is similar between the various models, even if each model highlights, obviously, a different experiential aspect of the Self.

For the sake of clarity on the knotty philosophical landscape about the Self, it is useful to categorize the different positions. On the one hand, we can bring perspectives together that consider the experience of the Self as a mental experience, caused by certain physiological constraints. These perspectives can be called constructivist or secretory^6^, because they outline experience as a sort of organic secretion (Miller, 1983; Searle, 1984; Pinker, 1997; Dehaene and Changeux, 2011), or as an internal computational construction based on the representation of the world outside us (Kircher and David, 2003). These approaches define the Self as an emergent causal property originating from the body, or rather from the complex interaction between brain, body and world^7^ (Dennett, 1992, 1996; Metzinger, 2003; Panksepp and Biven, 2012; Churchland, 2013; Gallagher, 2017).

Within CTS, we can differentiate various positions: functional constructivism, representational constructivism, semantic-conceptual constructivism, narrative constructivism. Each of these positions addresses different aspects of the Self, but they all share the same assumption that: the phenomenal result (the Self) is identical with the physiological functioning of the brain and it is identical with the scientific description of that physiological functioning. The discrepancy between phenomenal experience (for example, the experience of myself while walking on the beach) and the neural substrate, which causes it (motor and premotor areas), is explained as the expected result of the ontological difference between the physical and the mental world. Any constructivist theory addresses this double ontology, and all these theories contain this postulation inside them.

If CTS is a possible account of the Self, another viable approach can be represented by all those theories that, both in physical (in terms of neural correlates) and in phenomenal terms, consider the Self as the matrix, the essential feature of any experience^8^ (Kant, 1787; Zahavi, 2005, 2014; Thompson, 2007; Damasio, 2010). Such theory is close to the phenomenological approach, and it is supported by neuroscientists and philosophers (Damasio, 1999, 2010; Zahavi, 2005, 2014; Strawson, 2017).

Matricial theory of the Self takes sides against the constructivist assumptions and supports the phenomenal primacy of experience over the physical-epistemological one. The *giveness* of experience, the fact that experience is necessary for someone, constitutes the conceptual core that, for phenomenology, justifies using the concept of identity to talk about phenomenal experience (Zahavi, 2014). This claim seems to be justified by the assumption that, since experiences always belong to someone, so any experience intrinsically implies the existence of a subject to which this right experience is given. To clarify the essence of this subject, matrix of experience, is the goal of phenomenology, particularly of Zahavi’s approach which identifies it with the *giveness* of phenomenal experience, introducing the concept of *Formeness* (Zahavi, 2014). The *giveness* of experience, granted by the structure of the body, is considered what remains unchanged and identical, nevertheless we feel different experiences, *Formeness* is what structure the entire subjective, phenomenal feature of the experience itself.

Furthermore, because of the importance attributed to subject ontology, it is possible to appreciate two discrete theories in regard to the empirical–phenomenal models of the Self, i.e., essentialism and bundle theory approach.

Essentialism (or *haecceitas* theory) argues in favor of the real existence of the Self (Strawson, 1959). Nowadays, because of to the assumptions of neuroscience, the self is considered a mental entity generated by a physical system (the body of the subject). In virtue of the causal link between body and mental entities, the Self is supposed to be identical with the body (Churchland, 2013), or better, with the working brain. So, we can say that neuroscience defends a dualist-substantialist approach to the Self.

On the contrary, supporters of the bundle theory approach believe that the experience of the Self is a bundle of mental experiences, since they deny any importance of the alleged phenomenal ontology. According to this perspective, nothing exists in the physical world like the Self. There is no subject, and the experience of the Self is a mental illusion, or delusion, created by the functioning of complex physical systems which generate consciousness from complexity (Dennett, 1992; Metzinger, 2003). For these theories, which define themselves as strongly materialist, investigating the problem of the Self yields no compelling result, because we are concerned with illusory phenomenal entities, having a representational nature and generated by those very biological systems without any ontological or metaphysical characterization.

### *Physics-alism* and Fallacious Premises

Without following up on each of these approaches, in this section I analyze three common premises that rely on the various empirical–phenomenal models of the Self.

First of all, one can note that each model starts from the same premise about dualism: two kinds of ontologies exist. The former is what it is, namely physical things, which can be mathematically quantified; the latter is the domain of appearance, namely the phenomenal appearance of things, the taste of an apple, the smell of red wine, etc. It is fair to say that the ontological Galilean dualism is the source of all these philosophical-scientific approaches. The problem is that cognitive science does not justify this premise but accepts such a premise without questioning it; this happens because the prevalent approach in philosophy of science and epistemology has been dogmatic materialism (Whitehead, 1925; Manzotti, 2017a), or *physics-alism* (Strawson, 2008). Dogmatic materialism endorses the idea that abstractions, which we use to clarify how the physical world works and how it is structured, are what the world really is.

Cognitive neuroscientists enthusiastically approved *physics-alist* dualism because it enabled them to describe the world in mathematical terms, so in absolute terms, without the need to empirically justify the existence of the qualitative, phenomenal, properties of experience within their theoretical framework (Sellars, 1962), because they do not exist outside the mind of the subject. In this theoretical framework, the phenomenal, the mental, the subjective, are products of brain functioning, not of properties belonging to the physical world.

Taking the *physics-alist* theoretical model for granted, the risk is to misunderstand what constitutes the model itself with a truth about our object of analysis; that happens because of the adoption of the mathematical explicative model which, through the predictability of its conclusions, leads us to believe that what really exists is the model itself. Husserl (1923) was one of the first to give us this advice, many years before this debate. The same advice is made nowadays by Manzotti:

The fact that we use numbers to efficiently express what happens in the world should not lead us to believe that the world is made of numbers. Numbers are shortcuts to physical occurrences, but physical occurrences are not made of numbers, Manzotti (2017a, p. 179).

What exists for science is indeed the absolute object, the scientific image we can draw from the mathematical model that establishes the research itself. We can say, abiding by the analysis made by Whitehead about science and knowledge (Whitehead, 1925), that science wanted, following ontological dualism and disqualifying sensory perceptions, to achieve epistemic monopoly and the ontological truth about the nature of the world reality.

As we have seen, the assumption of ontological dualism within its own premises indelibly marks the philosophical-scientific debate on the Self; furthermore, this premise fosters another misleading argument, namely the prejudice of the subject. Many scholars, among them phenomenologists and neuroscientists (Damasio, 2010; Zahavi, 2014), claim that, because there is something like phenomenal experience, this must belong to a subject, since phenomenal experience necessarily belongs to a subject. Nevertheless, empirical reason does not exist to support this claim; there are not any phenomena that belong to a subject, rather the phenomena are relative to the subject.

We can say, favoring a neutral language free from the idea that a phenomenal ontology necessarily exists, that each experience is relative to the physiological condition of existence laid down by the body involved in that experience. Experiences do not belong to subjects but to bodies (which we call, detrimentally, subjects) and are relative to them. The concept of subjectivity is preferred by neurophenomenology compared to the concept of physical relativity, because it embodies the assumption that the point of view of the subject is the phenomenal one, namely what establishes the nature of experience itself. This assumption sticks to the prejudice of the subject (and to ontological dualism) and then to a *petitio principii*. Phenomenology should clarify what the subject is by means of argumentation. If it fails to do so, it shall be limited to an *a priori* postulation of the subject’s existence and it gives only a passive description of the different practical features of the Self with the aid of neuroscientific evidence about brain functioning.

The third materialist premise, which derives, necessarily, from the preceding premises, amounts to the idea that experience is identical with its physical condition of existence, namely the body. Nevertheless, the physical condition of existence for experience yields no information concerning what we are experiencing, but it only gives us information about how we are living that experience, how we can live it. Many scholars, following the *embodied cognition* approach, attribute the power to cause phenomenal experiences to the body (and to the brain) which allows us to have experiences, and therefore they attribute the identity of certain cerebral states (namely the neural correlates of experience) to the phenomenal experience of existing.

Despite this, to an authentically pre-reflective approach, free from philosophical prejudices, the alleged phenomenal character of experience defended by neurophenomenology and attributed to the body, seems to be only a linguistic and conceptual description of relative existence. Relative existence is something physical, which is allowed by the different physiological conditions of existence that are inherent in our bodies, but it is different from the body.

According to our perspective, *Physics-alism* relies on assumptions conveyed by conceptual prejudices that, since they are misleading, cause false conclusions contrasting with our goal, and prevents us from describing experience in a completely naturalistic way.

## Neo Naturalism: Premises and Arguments

It is possible to escape from the vicious circle of *ad hoc* explanations defended by physics-alism through a critical analysis of science abstractions (Whitehead, 1925) enabled by the adoption of a real and mature Naturalism. This kind of Naturalism is guided by the need to make a critical review of scientific assumptions in light of the comparison with the reality of experiences.

We can call this perspective Neo Naturalism. According to Neo Naturalism, there is no need to postulate any *ad hoc* explanation, built on the models which we want to justify, to describe what experience is. A Neo Naturalist approach is, therefore, the attempt to propose a purely physical theory of existence that is able to balance our knowledge about the nature of physical objects, reality, the role of the body and the nature of the experience itself. The Neo Naturalist perspective offers the occasion to exchange our bad premises, which are the basis of *physics-alism*, with logically and empirically evaluated arguments.

The starting point of Neo Naturalism is to deny the Galilean ontological dualism. We have already underlined how the subscription of this premise is totally unjustified at an empirical level, but it is widely accepted as a fideistic duty (Whitehead, 1925; Strawson, 2008; Manzotti, 2017a). For this reason, Neo Naturalist models line up against the hallucinatory models of perception, which have a great charge on the creation of the empirical–phenomenal models of the Self, particularly against disjunctivism (Hinton, 1973; McDowell, 1982; Byrne and Logue, 2009). Ontological dualism implies a misleading conclusion: confusion between experience and knowledge. Hallucinatory models of perception are built starting from such idea: to be truthful, namely a sure source of knowledge, perception should be able to gain absolute knowledge about external objects. These absolute properties are identified with the mathematical structure of objects. Nevertheless, since the idea of absolute property, or primary property of the object is a chimera, an idea without any empirical validation, it is hard to defend an argument, and an explanatory model, based on this idea. Hence, Neo Naturalism explaining the hidden premises behind that, makes this argument less rational than its defenders would like.

In contrast with hallucinatory models, *Neo Naturalism defends a realist account of perception and experience*. As Manzotti claimed (Manzotti, 2017a, p.188^9^), experience is not true or false by itself, experience simply is. *Experience actualizes the existence of an external object property inasmuch it is relative to our body* (which we can call: *actualizing object*). Experience is different from knowledge, but they are not totally separated. Knowledge indeed, inasmuch as it is abstract, find its justification into experience: from sensory data we can draw conclusions about the different relations between various phenomena; it is through the occurrence of functional relations that we learn how things take place, we learn to know the causal structure of nature (Manzotti, 2017a, p.189). Therefore, defending the epistemic and ontological dignity of sensory data, we can talk about, for a good reason, the ≪incorrigibility of experience≫, which derives from the ≪principle of the incorrigibility of existence≫, (Manzotti, 2017a, pp. 191–193). *Existence indeed is not committed to the truth, experience is about what exists, it is not about the way in which something exists, in the same way perception does not leaves us any truth about how things work in the world, rather perception gives us the world, or better the part of the physical world relative to our physiological existence conditions*.

From the Neo Naturalist perspective, hallucinatory models of perception decay; Neo Naturalism claims indeed that *perceptual errors do not exist at all, because we can experience only actual objects, objects relative to our bodies, hence existing objects.* The error is entirely conceptual, namely it regards the linguistic limits we have to report it, not the experience itself. For example, we can be wrong about gastric pain etiology, but we can’t be wrong about the pain we are feeling now. This impossibility of error is not due to our alleged privileged epistemic authority (Shoemaker, 1994) but it is due to the incorrigibility of perception, which presents us the world physical configuration that takes place relative to the physiological existence conditions posed by our bodies.

Talking about perceptive errors is hence inappropriate ^10^, as it is inappropriate to formulate models of perception based on the illusion argument (Russell, 1912; Austin, 1962). Errors are contained not in to the perceptive event, but rather they are contained in the categorical synthesis which we make. In other words, errors appear from the observation of certain perceptive *datum*, which is not in itself true or false, right or wrong, it simply is. So, perception can be a knowledge tool, but it does not give us, necessarily, the absolute knowledge of the object itself; perception gives us only knowledge relative to the physiological conditions of existence of the body involved in that experience, giving actual knowledge to us about the object of experience. This means that perception presents us with the objects which populate the world in an identity relation. The relative existence of these objects is what constitutes our experiences.

By questioning ontological dualism, it is possible to undermine another assumption endorsed by *physics-alist* models of the Self, namely the prejudice of the subject.

Neo Naturalism highlights the dualistic weight on the separation between subject and object. If, at this point, we have not any argument to empirically support the existence of phenomenal ontology, it is unclear how *physics-alism* can claim that the subject perceives different properties, phenomenal or qualitative, as compared to the mathematical properties presented by the absolute object. The only argument presented in defense of this position is anthropocentrism, largely widespread in scientific and humanistic culture; it is what Manzotti called ≪subject dependence≫ (Manzotti, 2017a). Human beings are indeed not inclined to lose their preeminent role, we want to remain *sui generis* compared to the physical nature of the other objects (bodies) which inhabit the world, even if this implies the support of such an irrational argument grounded on a *petitio principii*. Giving up the subjects’ preeminent state is hard because it means giving up many old ideas such as subjectivity, phenomenal experience, non-physical existence of something, like mental states or mental representations. It means to give up our privileged, ontological state to became equal to all other objects in the world, it means to be equal with rocks, trees, stars, dogs; it means to get off our high horse of anthropocentrism to return for being part of the physical stuff of the world again.

Hence, Neo Naturalism suggests replacing the concept of phenomenal experience with the concept of relative experience. All the objects in the world have many relations with other objects, this situation is described by physics with the term “relativity.” All objects indeed, microscopic or macroscopic (atoms or planets), are in a relative relation (namely a relation influenced by the physical condition of existence) with other objects. To explain this relation in physics it is not necessary to bring into play the notion of subject, because the subject, with his physical capacities (mental, or conceptual capacities are, for Neo Naturalism, entirely physical) demonstrates only the conditions of existence for a certain experience of an object X. The object X experience is not identical with the conditions of existence (the bodily constraints) which allow the experience itself, rather it is identical with what it is: the object X, or better, the relative object X.

To understand the Neo Naturalist approach and the Spread Mind Theory it is necessary to describe the idea of relative object.

The idea of absolute object supported by dogmatic materialism is the idea about an unreal, timeless, motionless entity. The absolute object is a scientific (mathematical) abstraction of the real object (Manzotti, 2017b); it appears to be unreachable through perception because perception is a physical mechanism and it perceives only physical things, physical objects, not mathematical or mental abstractions. For this reason, none of us has ever seen the length of objects, or no one has ever perceived the temperature of a sunny day in Celsius degrees. We are not built to perceive mathematical properties of reality, we are built to perceive all physical properties of reality which can take place in relation to our physiological conditions of existence. We believe that mathematical properties constitute objects in themselves because we trust disproportionately in our abstracting capacity. The idea of absolute objects has been accepted since the era of Galileo, and continues in our days, as a consequence of a fallacy which Whitehead called the ≪fallacy of misplaced concreteness≫ (Whitehead, 1925; Manzotti, 2017b). Science, indeed, believed that abstraction can be considered as things of the world, as real entities. Despite the faith that science gives to this intuition, there is not any empirical proof, or logical argument that lets us decide on a positive conclusion about the physical existence of scientific abstractions. For this reason, the absolute object postulated by *physics-alism* appears to be an umpteenth entity created to give epistemic and ontological value to science herself, rather than being a real object of the world.

Objects which populate our lives are not dumb or without any meaning, they are ≪active, relative, causal and temporally constituted≫ (Manzotti, 2017b). It means that the objects which we experience in our lives are not abstract mathematical models, rather they are objects which exist relative to the causal contingent circumstances offered by our bodies. *Relative objects indeed do not exist independently from other objects (in the case of experience they do not exist independently from our bodies)*; they are not eternal entities, because the relation between our body and the object is temporally and spatially determined and hence physically determined and active. *The relation between two objects is a mutual ‘taking place’ of the existent. Therefore, actual objects are real physical objects, and the existence of these objects depend on the ‘taking place’ role of our body. Our body, however, does not constitute experience but is restricted to the contingent causation of experience, since it offers the right physical circumstances that enable an object to ‘take place.’*

A physical example which can help us to understand the idea of actual object and the idea of relative object and reference object is the notion of speed and the notion of reference frame. As stated by Manzotti ≪the body is a complex reference frame. It plays just the same role of a reference frame for relative velocities, only it is much more complex≫, (Manzotti, 2017b, pp. 42–43). The experience of certain object properties (colors, sounds, smells), like speed, is the result of the relation between the object of experience and a certain reference structure (the body) which can actualize the object’s proprieties. Experiencing colors for example, like speed, is just a physical relative experience which implies the existence of two physical objects. No object is characterized by absolute speed, it always has a certain velocity that is relative to a certain reference structure, exactly in the same way we have argued before for an object to have properties (colors, smells, sounds, etc.). *No object is an absolute entity in itself; the physics-alist idea of absolute objects is a Platonic chimaera, not a physical reality. Real objects are, like speed, entities belonging to the physical world, and they happen, they exist, only when another physical object is connected to them, because the actual structure of such objects constitutes the actual properties of other physical objects*.

Let us make a practical example: I am on the motorway which links Rome and Milan, and I am driving my car at the speed of 120 km/h. I am in the fast lane, on the side lanes there are two cars: the former, on the right, is traveling at 110 km/h (car A), while the latter is traveling at a slower speed, 80 km/h, on the fast lane (car B).

My speed is 120 km/h; nevertheless, its rate changes if I take as reference framework not the ground, but other cars. If the reference frame is car A, my relative speed will be 10 km/h, instead, if the reference is car B my relative speed will be 40 km/h. If, at the same time, there is a hawk flying in the sky above me, at the speed of 20 km/h, my speed, in this case, will be 100 km/h.

Speed is a physical property. Car speeds are all physical and at the same time they are all private, or subjective properties, according to the *physics-alist* idiom, because all of them refer to a certain reference framework, the ground, car A, car B or the hawk. In other words: only a specific reference framework (a certain object, or body) can actualize a certain car’s speed (that is the actual object or relative object).

According to SMT, experience, being a physical thing, acts in the same way as speed. Therefore, it is a physical object which is in a certain space at a certain time; this object is not a Platonic or Galilean absolute object, rather it is a relative object, because it is contingently caused by the physical existence condition imposed by the actualizing object (the concept of reference frame used in the case of velocity is identical with the role of the body in the case of experience). We must be very wary in order to not be deceived about the physical nature of experience. It is necessary to avoid mistaking physical properties, like speed or experience, which are the physical object itself, for the mathematical quantification, or the conceptual description of this property (speed X, color X, smell X, etc.).

Furthermore, Neo Naturalism offers an alternative solution even for the third *physics-alist* assumption: the identity between the body and the subject.

Thanks to the dominant scientific approach in the philosophy of mind debate, one can appreciate the re-emergence of dogmatic materialism regarding the actions of the body fostered by embodied and enactive theories. The body has become the new aprioristic reference to those who want to dogmatically investigate the nature of experience, so it is then considered the right physical entity to explain what experience is, using our conceptual structures, in this case the neuroscientific ones.

Nevertheless, if we start asking questions about individual identity, in the biological domains, we will see our aprioristic certainties falter. Biologists indeed need expanding on many issues concerning the organism identity, so we can appreciate how they had shifted from a dogmatic description (the concept of immunity as internal function of the body which generates immunity itself, just to give an example), to a Neo-Naturalist approach of organism identity. These kinds of research show us that, similarly to the experience of ourselves, our body is identical with the immanent experiences which it has with the world (Pradeau and Carosella, 2006; Tauber, 2013; Gilbert and Tauber, 2016; Pradeau, 2016). The body is not identical with something postulated or derived from abstraction; in the case of biology, the experience of what we talk about is, for example, an inflammatory experience, because we are talking about immunity. Nevertheless, in both experiences, immunity or sensory experience, what constitutes the experience (what is identical with our experience) is not the idea of immunity, or the Self (namely the abstract principles which we postulate by observing the body’s physiology) posited by us, but the inflammatory experience itself, the pathogenic agent in the case of immunity, the relative object in the case of one’s experience.

The body, similarly to the experience of ourselves, is not something we can find inside us, or reflecting upon what we observe about bodily functioning; it is something that we live through experience and it is identical with it. Our body is not identical with its biological functions postulated by body physiology. Organisms are structured by causal processes, namely organisms are causally constituted by all the relative objects to which physiological structures give the occasion to take place.

Hence, the identity between the body and the subject, supported by cognitive neuroscientists and philosophers, seems to be unjustified, there are no empirically justified arguments to support these conclusions. If we look inside the brain, or inside our body, there are not any experiential features. We only find neural cells which do their work, activating in accordance with the received input. Therefore, it seems that the physical correlate of experience proposed by cognitive science, the brain, is not able to explain the issue of experience through identity.

It is possible that we are searching for the answer to the “hard problem” in the wrong place, (Dennett, 1992, p.109; Manzotti, 2017a, pp. 26–30), namely in the brain. The brain indeed was chosen as the best physical candidate only out of mere suitability, because it is a defined research target about which neuroscience gains more and more data; the brain is a well-known object on the basis of which are made many mathematical models that allow neuroscientists to arrange experimental sets and build theoretical frameworks. Nevertheless, such prejudices are not compatible with the empirical reality of things (Manzotti, 2017a; pp. 26–30).

*There exists another candidate, equally physical, which has exactly the same properties of our experience: the external object which constitutes such experience.* SMT, endorsing a Neo Naturalist approach, supports this idea. In the case of the visual experience of the oak, for example, what constitutes the experience, what is identical with the experience, is the tree itself, the actual oak, the oak pertaining to the existential conditions posed by our body; the actual object, or the relative object exhibits all our experiential features. *The body is the contingent cause of certain specific experiences, but it is not identical with such experiences. Cortex properties indeed are different from that experienced in the experience of the oak. What is identical with the experience is the object which exists thanks to our body* (the experience of the oak is identical with the relative physical oak perceived by my body), this object is the relative object (Manzotti, 2017a).

## Neo Naturalist Bundle Theory of the Self

The Neo Naturalist theory defended here, which I call the NNBTS, aims to provide alternative approaches to those that have been defended by *physics-alist* approaches by relying on three main premises. I shall outline these three ideas:

(1) NNBTS, according to Hume, supports the idea that *personal identity is not a metaphysical or conceptual construct, but it is a bundle of all our perceptions* (Hume, 1758), which are our first source of knowledge. Nevertheless, against Hume’s opinion, NNBTS dos not support radical eliminativism. Asserting that we are identical with our experiences does not equate with the conclusion that the Self is an illusion, an unreal experience; rather it means to consider the experiential reality itself, namely to go toward the world and the objects that live in it because they are what causally constitute experiences^11^.
(2) NNBTS defends the idea that it is necessary to describe experience at a pre-reflective level. NNBTS, abiding by Neo-Naturalism, projects itself as an ontologically democratic, realist and minimalist theory, which tries to avoid the use of *ad hoc* explanations and conceptual dogma to explain what the bundle of the Self is. This is the reason why *NNBTS aims to clarify experience in its pre-reflective dimensions of existence because it is the authentic dimension of relative existence*.
(3) Furthermore, NNBTS, following SMT, makes claims in favor of the experience reality, against Galilean ontological dualism, arguing that experiential reality is not an internal subjective condition, identical with alleged mental phenomena belonging to the subject, or to his/her cerebral functions. *Experiential reality is identical to all the physical properties of the object of perception, it is not an illusion or a virtual reality, it is the part of physical reality which we are identical with*, given certain physical and physiological conditions of existence.

*NNBTS claims, defending a sparing, physicalist and realist approach, that the experience of ourselves is identical with all physical objects which constitute the bundle of our experiences.* In other words: identity is not between a postulated subject and her subjective mental representations, neither between subject and the alleged phenomenal character of experience, or between subject and neural activity, but it is between experiences and the objects of these experiences, they are identical among them because of their immanent relation, their relative existence.

Through the adoption of Neo Naturalism, all the premises of *physics-alist* models are overturned: *ontological dualism is replaced by ontological monism grounded on the physical nature of world objects. Neo Naturalism stops relying on circular arguments and changes into a simple and clear argumentation intended to avoid ad hoc explanations built on the obtained data*. Finally, *the unlikely identity between body and experience is reconsidered in the light of the physical world, attributing its worldly foundation again to experience, and giving to the body its causal contingent role of actualizing object.*

### Neo Naturalism and Empirical Evidences: The Case of the Rubber Hand Illusion

The Neo Naturalist model of personal identity defends the causal and experiential role of the body, but it discusses the aprioristic and absolute role attributed to the body by cognitive neuroscientists, questioning the proposed identity between us, our phenomenal experiences, and body functioning.

As we have seen, empirical–phenomenal models of the Self are built on the postulated idea of subject and person. By doing so, they support the idea of a subject self-confined in his own mental and conceptual world, and also confined within his own body, which generates phenomenal experience as mental representations. Embodied cognition shares the idea that we are identical with the body-brain functioning, and its proponents justify this idea by referring to the notion of body ownership:

Body ownership is a special perceptual status of one’s own body, which makes bodily sensations seem unique to oneself; (Tsakiris et al., 2006).

The sense of body ownership is the feeling that “my body” belongs to me and is ever present in my mental life; (Gallagher, 2000).

Body ownership is a phenomenal experience allowed by two kinds of representation, namely body schema and body image. Body schema is a dynamic action-oriented bodily representation (De Vignemont, 2010) which is still at a sub-personal level of consciousness; while body image, built on this first proprioceptive representation of the body, is conscious and allows us to identify ourselves with our body and its actions (De Vignemont, 2010; Tsakiris, 2010).

We can easily say that the cognitive model is a representationalist model of bodily experience, so it is hallucinatory, exactly as the empirical-models of the self, namely based on the assumption that we create a mental image of our body. So to speak, this kind of model defends a Kantian perspective about the role of the body that is considered to be the representational center able to link subject and world. The subject indeed perceives the world outside of him/her starting from the representational model of the body; this process generates the phenomenal experience to be our body. In this way, the body becomes the matrix of all experience.

Starting from these assumptions, neuroscientists have been using perceptive illusions about the body as a paradigm to understand how bodily perception works. This idea is admitted to even by the fathers of one of the most used paradigms, the Rubber Hand Illusion paradigm^12^, which say: ≪Illusions have historically been of great use for psychology for what they can reveal about perceptual processes≫, (Botvinick and Cohen, 1998).

In the RHI paradigm, for example, the illusion of perceiving the rubber hand like it was our real hand, offers the occasion for the neuroscientist to study how a representation can be construed. Let me sketch the RHI experiment briefly. The subject’s real hand is hidden under a table, or behind a screen. Instead of the real hand, a rubber hand, very similar to the real one, is put on the table in front of the subject who is requested to look at the fake hand while it is being touched by a paintbrush. At the same time, even if hidden to sight, the real hand is touched, synchronically with the fake hand. In this experimental situation about 80% of the participants report to have felt the tactile sensation not over the real hand, but on the rubber hand, as if they had have incorporated the rubber hand inside their bodies (Armel and Ramachandran, 2003; Tsakiris and Haggard, 2005). This result allowed neuroscientists to say that we are able to build illusive bodily representation based on the source of sensory and proprioceptive inputs (Tsakiris, and Haggard, 2005; De Vignemont, 2010).

There is a postulated property in the RHI paradigm, exactly as in other hallucinatory models of perception. Such a property is the alleged standard bodily representation - body schema and body image- of our biological body, which justifies the use of the concept of illusion. In the case of RH, because we do not have a rubber hand attached to our body, the phenomenal experience of feeling the rubber hand, like a biological hand, is, for neuroscientists, clearly the product of an illusory bodily representation. But, if we endorse a different theoretical framework, as Neo Naturalism is, will we obtain the same data and conclusions?

If the existence of an innate physiological body schema based on the causal connections which links the body and the brain (Melzack et al., 1997) is acceptable, it is difficult to justify the physiological existence of phenomenal bodily representations because we should prove the identity between our bodily representation and our bodily experience. However, they are clearly not identical, because representations are not physical things. On the contrary, due to the adoption of the realist model of perception defended by SMT it is possible to revisit the RHI and speak about the Rubber Hand Experience. It is not only an interesting reading of the RHI experiment, but it is also an empirical test for the Neo Naturalist Model of the Self.

SMT claims that there is identity between our experiences and the physical objects which cause those experiences, for this reason it defends a completely realist and direct theory of perception (see Manzotti, 2017a). In other words, each an experience, even those defined as hallucinatory or illusory, is identical with something physical which caused it. This enables us to avoid using the concept of illusion, which is only a concept and not a physical state of things (see p. 3). Manzotti distinguishes between three kinds of perception:

The first class is every day perception. One perceives things as they actually are […] we experience a red apple because our experience is the red apple. The second is the case of illusions. One perceives something differently from what it is. More correctly, one believes that one perceives something differently from what it is. For example, I perceive an apple as red but have reason to believe the apple is green. […] Finally, we face the huge class of hallucinations. I propose to divide them into ordinary and extraordinary hallucinations. Ordinary hallucinations are experiences of objects or of parts of objects that one has experienced before. Extraordinary hallucinations are experiences of objects or parts of objects whose components one has never experienced before (Manzotti, 2017b, p. 128).

The SMT taxonomy of perception allows us to clarify the case of illusory experiences, like RHI, in a realistic way. The sensations of feeling the tactile stimulus on the rubber hand, and to own the rubber hand, etc., are not illusions, they are wrong beliefs conveyed by our perceptive habits. Indeed, we are familiar with our body, which is the object that we perceive all the time, and with its movements; though in the experimental situation, where an object similar (in shape, color, spatial placement, etc.) to our body, in this case to our hand, is presented, we are persuaded to justify this perception in line with our usual perceptive experiences that are influenced by our retrospective inferences. Indeed, every time we perceive an object which has this particular shape, or color, or spatial placement, we are perceiving our hand; furthermore, in the experimental set the synchronous stimulation between the rubber and the real hand is fundamental to elicit the result, because proprioceptive feedback actualizes the usual experience of the real hand with the visual perception of the rubber hand in front of us. In this way it is possible to integrate this unusual experience into our actual experience, creating the strange feeling of being an object that is very similar to our hand, but it is not our hand, it is not a part of our body.

This strange experience, contrary to what neuroscientists would say, proves exactly what SMT and Neo Naturalist Model of the Self, claims: *we are not our body, we are world, we are the objects which constitute our experiences, even in those particular cases in which we have unusual experiences that do not enter into our habitual category and that, for this reason, we label as illusory.* In the case of RHI, the chimeric, or unusual experience, is that of perceiving the false hand as a real hand, together with the sensation of touch on the real hand identical with the tactile stimulation seen on the false hand. Because the rubber hand is placed at a certain distance from us and stimulated synchronously to the stimulation of the real hand, such experience is integrated into our usual belief about the body since, usually, when we have this kind of experience, we are perceiving our biological hand. The same idea is defended by Bill Brewer, who states that:

Bodily ownership is experienced in this extension of the subject of experience into the material world. The peculiarly intimate sense in which my body-parts seem to be mine is just that in which they seem to be parts of the spatially extended physical body which I seem to be. Experienced bodily ownership, then, is awareness of oneself as extended in space (Brewer, 1995).

Concluding, starting from the realist model of perception, defended by SMT and Neo Naturalism, it is possible to change the bad premises shared by hallucinatory models about the special ontological role of the body in generating phenomenal experience. SMT and Neo Naturalism highlight the prominent role of the body in experience by saying that it is not a special object among others, rather it has a double causal role. *The body is both an actual object (like an apple or a tree) and an actualizing object, namely it is the condition of existence, or the reference frame, for the actualization of certain objects*. The RH Experience, for example, is constituted both by the body as actual object (real perceived hand) and by the body as actualizing object for the rubber hand, like a body part because similar to it. The body has a paramount role, since it is the central component that allows experience to take place. It is in virtue of the causal structure of the body that one has the chance to actualize the world. However, the body only defines the way one can actually perceive an object, without saying anything regarding the actual experience of it. Hence, the identity between body and experience, or between brain and experience, supported by cognitive neuroscience, is misleading, because it mixes up what causes experience at a contingent level (the body-brain) with what experience is (the relative object). The Neo Naturalist approach brakes with this solipsistic tradition and through a physical theory of experience aims to bring back the subject into the world. The Neo Naturalist Self opens up to the world, it sidesteps the abstract schemes of materialism and comes back to be a part of the great book of the world (Descartes, 1637). Experience and the world constitute indeed our first source of knowledge: no thought can exist without a physical experience able to cause it, no sensation or memory can move us if it is not a real one. By real I mean caused by a physical, real and authentic object.

### Neo Naturalism and Person

The problem of the subject is historically linked with the concept of person grounded in the faculty of memory. This definition is dating back to Locke, who said that a person is ≪a thinking, intelligent being, that has reason and reflection, and can consider itself as itself, the same thinking thing, in different times and places≫, (Locke, 1694, p. 335). Nevertheless, according to some critics of this argument (see, Perry, 1975), the memory criterion is the starting point of the model of psychological continuity. This perspective states that our personal identity is grounded in the identity between us and our mental states (that are representations) which, being in a causal relation among them, are what is necessary and sufficient to guarantee the persistence of the subject through time (Shoemaker, 1970; Nozick, 1981; Lewis, 1986). This perspective defends an internalist and representationalist account of memory. As we have seen, all models of perception are hallucinatory and exclude the external object from the causal chain which generates the experience itself; the same is true for memory: memories are generated by spontaneous brain activity, they are considered mental objects, not physical ones.

Furthermore, the memory criterion is based on the acceptance of the idea of absolute Newtonian time. For mainstream approaches, time is like a straight line where we can find our experiences as points. Something that happened to me yesterday is located, on the timeline, on yesterday, and I can re-activate this experience by fishing out the correspondent mental states which contain my memory of this past experience. In this way, time is a kind of label for our experiences, it is not something existing, it is only a linguistic category where we can put in our experiences on the basis of our bodily proximity with the event itself. Usually, when we have just 150 milliseconds of time span with an event (or better with an object) we speak of perception and we locate it in our present, if an event is 1 day, or 1 week, far from us, we speak about memory, locating the event in the past.

On the contrary, Neo Naturalism rejects the hallucinatory model of memory, preferring a realist account in analogy with the realist model of perception proposed. To defend this account the first step needed is to analyze the concept of time which is implicit behind the hallucinatory models of memory. Indeed, as claimed by many authors as Bergson (1896), Manzotti (2017a), Rovelli (2017), and Einstein (1916), the idea of absolute time is physically incongruent because we cannot perceive abstract time, we cannot perceive time as fixed point, we perceive time through the change in objects. The idea of absolute time is akin to the idea of absolute object and, exactly like that, it appears to be empty, it is an idea which collapses in comparison with physical reality. Absolute time cannot exist, it is only our abstraction. Maybe it can be useful for our practical goals, nevertheless there are no reasons to justify this abstraction, as highlighted by Manzotti:

There is no physical law dictating why the time span of the present should be less than a certain value. In fact, either the present is punctual or it isn’t. If it wasn’t, it should be smaller than a certain Δt^∗^, Δt < Δt^∗^, but why could there be a critical value? Why should Δt^∗^ not be arbitrarily large – 10 ms, 100 ms, 1 h, 1 day, 1 year, 1 billion years? […] Nothing in physics dictates a minimal time span. Thus, the notion that the present is more or less what happens now is irredeemably vague. (Manzotti, 2017a, p. 111).

For this reason, Neo Naturalism defends an idea of time as a feature of experience, as physical object. The present, as the past, should be considered as a whole of existing physical objects which have a causal relation with us (Manzotti, 2017a). This causal relation establishes what we usually call our present, or our past (reenacted in memory forms); in other words, it establishes our experiences bundle, what we call our Self.

Starting from this alternative conception of time, the psychological continuity view assumes another value because it is useless to maintain the representational account of memory. From a Neo Naturalist perspective, memories work as standard perceptions: the memory of an object X is identical with the object which caused this memory, this experience. Hence, memory is a reenacted simple perception, namely it renews the causal link that the object had with our physical body, bringing the object in our present again.

Let us take a practical example. I remember a day spent with my grandmother making pizza, I categorize this event as past, happening 5 years ago. Adopting the Neo Naturalist perspective, the memory of my grandmother while we were preparing the pizza, the flavor, the water, the yeast, are actual re-actualizations of those objects which, in the moment they “bring to mind,” they are my present. The same idea is defended by Manzotti:

One experiences the past to the extent that the past is still present. Memory is the delayed, yet ordered, perception of the past […] I experience the past, what is called the past is part of the present. Memory is nothing but identity with a past that is still present. (Manzotti, 2017a, p. 196).

There is a classical objection for the realist model of memory. It regards the fact that we do not have an exact memory of our past experiences. This objection sounds like this: if we endorse a realist model of memory and perception, the link between experience and object is necessary direct, so how to explain our bad, or partial memories? Mainstream models explain this fact as a matter of representational construction of past memory, creating the idea of a subject which generates his/her memories in a continuous narrative of his/her past and of himself/herself.

On the contrary the Neo Naturalist answer to this question is very simple. Why is my memory not identical with my original experience? It is because the conditions of existence for the re-actualization of such experience are not the same. My body indeed is not the same of 5 years ago, furthermore I am not at the same space-temporal distance in respect to those objects, of that physical reality. Nevertheless, in the moment in which my actual perception offers the occasion to actualize that past event again (for example a pizza that tastes like my grandmother’s pizza), such an event is my actual present, namely it is the actual object which I am perceiving now.

The body has a paramount role because it is the physical object which actualizes the causal connection between a past event and our present body; in this way the body re-activates the past reality into the present one. The body is a part of the causal chain which has actualized the original event, so it can re-actualize such event many times again, only with different conditions of existence because it constantly changes.

## Objections

The NNBTS faces many philosophical objections; let me consider one of them: the nature of the bundle of personal identity.

In this paper, I’ve tried to define the concept of Self-identity through the idea that what is identical with our experience of being ourselves, what constitutes this experiences bundle, are the relative physical objects which cause such experiences. Claiming that, it is necessary to confront the Special Composition problem (Simons, 1987; Van Inwagen, 1990; Varzi, 1996; Merricks, 2001). The constitution problem is indeed one of the most debated ontological issues (Simons, 1987; Van Inwagen, 1990; Husserl, 1901/1902; Varzi, 1996; Sider, 2001; and the binding problem in neuroscience, Chalmers, 1996; Engel and Singer, 2001); however, address this problem is beyond the scope of the present work, what I want to do here is just to clarify the concept of constitution in order to avoid a philosophical misunderstanding on this key point.

There are three classical approaches to the composition problem: universalism, nihilism and restricted composition theory. Universalists claim that parthood involves the emergence of infinite numbers of wholes, because they state that whatever two things there are, then there is a third thing which is constituted by the two (Lewis, 1986). Nihilists, instead, claim that there are not wholes at all because, as stated by Van Inwagen, ≪It is impossible for one to bring it about that something is such that the xs compose it, because, necessarily (If the xs are two or more) nothing is such that the xs compose it≫, (Van Inwagen, 1990, p. 72). Finally, the third option is the Restricted Composition theory which states that there are limited numbers of wholes in strict relation to our linguistic and conceptual ontologies. Such approaches are all logically respectable, but they don’t fix a physical well-defined criterion to decide when a thing constitutes a whole and how it is constituted by its parts, so they remain vague and there is the lack of possible empirical validation (Manzotti, 2009). For those reasons mereological thinking alone seems not to be enough to solve the constitution problem (Varzi, 1996, p. 10).

There exists another possible approach to that problem and it can help us to establish a physical criterion to clarify the constitution problem: it is the Causal Composition Theory developed by Manzotti (2009). This theory tries to eliminate the vagueness, and the conceptual arbitrariness of Universalism, Nihilism, and Restricted Composition by introducing the joint causation criterion to identify what physically constitutes a whole. The argument which supports the Causal Composition Theory is structured as follows:

P1. Since we are in a physical world, we must adopt a physical explanation of the existence of things.

According to many authors (Merricks, 2001; Kim, 2005; Manzotti, 2009, Manzotti, 2017a) “to exist is to have causal powers.” In the physical world it implies that “to be is to be the cause of a causal process” (Manzotti, 2009). This leads to a second important premise that states:

P2. Something exists *iff* it is involved in a causal process, or better: something exists *iff* it is the cause of some causal space-temporal process which produces some space-temporal effect^13^.

We have the possibility to make important conclusions about the notion of existence and constitution from those two premises about the causal structure which establish the physical world. The first claims that:

C1. Physical existence is relative because to exist implies being in a causal relation with something else, namely to exist implies joint causation. This causal relation is in fact a physical causal process which crosses time and space (Manzotti, 2009).

This first conclusion argues that constitution is a matter of causal processes and not of mental constructions, conceptual emergencies or eternal essences. In the physical world what exists is constituted by chains of causal processes which take place in a certain space at a certain time- so to speak, an existing object is made by all causal processes which concern it. This conclusion is fundamental because it eliminates the arbitrary idea of eternal object or objects with an internal *haecceitas*, with intrinsic essence; C1 gives us a fixed criterion with which we can say empirically when an object constitutes something else, it is physically justified, and it matches with the proposed idea of relative objects. Furthermore, C1 guides us to the conclusion (C2) about what constitutes the Self bundle; the joint causation idea indeed enables us to physically identify what constitutes the bundle itself. The causal composition argument allows me to make the second conclusion which better explains my claim that we are identical with our experiences bundle:

C2. The whole Self-bundle is constituted by all those physical objects which are in a causal spatial–temporal relation with our body, which is itself responsible of a causal chain to some in relation to other physical objects.

Then, when I claim that our experiences are identical with all the relative objects which constitute such experiences, I mean that they are causally constituted by those objects. So we have a physical criterion to describe when something constitutes something else and what kind of relation it implies, namely a causal space-time oriented relation.

Causal Composition Theory offers us the possibility of preventing the worthless breeding of entities made by Universalism because it offers the occasion to avoid the aprioristic, timeless and spaceless existence of objects; furthermore, CCT helps us with the problem of negative proliferation made by nihilism, blocking our tendency to deny what we are not able to understand and offers us a physical solution to stem the conceptual vagueness of the Restricted Composition theory. For what concern NNBTS, CCT helps to establish the boundaries of the Self bundle, identifying the causal processes chains which are identical with the bundle itself, and clarifying the concept of constitution which is beyond that theory. Constitution is not a conceptual term anymore; rather, constitution seems to be a matter of physical entities which are engaged in a causal relation, having certain space–time coordinates. This relation in fact makes them what they are. To conclude, what constitutes the experience bundle is the part of physical reality which exists in relation to our body, which is a causal object as many others in the world. Objects are causes for the existence of the Self bundle and for that reason they constitute the bundle itself.

## Conclusion

I contended here that we are identical with the physical objects that we can perceive in virtue of our physiological reference frame, the body. Neo Naturalism states the concept of identity in a non-tautological way, namely highlighting how the subject is identical with the objects which constitute her experiences. Neo Naturalist identity dwells on real, actual, relative entities, rather than on absolute, postulated, tautological ones collocated in an ideal world. So, relative objects constitute the Self experiences bundle; they are existing, they are in the world, they are colored, scented, tasty or smelly, but most important: they are relative to our bodies. The result of the explanatory undertaking is an existing, physical, actual subject: a subject willing to accept changing shapes, changing experiences inside of herself; a subject which, free from all his conceptual residues, does not have a problem to embrace her *status* as object among the other objects of the world. The subject which Neo Naturalism leaves us, is chimeric and manifold. It is a subject that learns to be an object among the many in this world. The Neo Naturalist Self loses its absolute existence to gain the reality of the physical world. This is the consequence that we have to deal with, if we want to outline a physical theory of the Self: the breakdown of our prejudice to be the only ones, different from the rest of the natural realm. In the physical world in fact, what we call subjectivity, because of our prejudices, it is simply relativity, without any hierarchy. The physical world is a community of objects that exist because of their interactions (Manzotti, 2017a). Luckily, this democratic and authentic world is what establish as ourselves, and it is real; indeed identity is not a conceptual or representational construction, but it is a physical reality, constituted by the physical world that exists thanks to our bodily conditions of existence, like a sort of mutual immanence dance (Whitehead, 1920). As Whitehead claims, identity is nothing more than the successions of our possibilities to have an experience, a dance between body and world, a dance between mutually necessary objects.

## Author Contributions

LC wrote all the manuscript sections.

## Conflict of Interest Statement

The author declares that the research was conducted in the absence of any commercial or financial relationships that could be construed as a potential conflict of interest. The reviewer AC declared a shared affiliation, with no collaboration, with the authors to the handling Editor at the time of review.

## Footnotes

1 In this paper, I will refer to the proposed theory, the Neo Naturalist Bundle Theory of the Self, using the acronyms NNBTS.

2 I will refer to The Spread Mind Theory using the acronyms SMT.

3 Galilei (1623). Husserl, in his latest works, clearly insisted on the role Galileo played in the development of ontological dualism (Husserl, 1954).

4 In this paper, I will use the words consciousness, experience and subjective or phenomenal experience as synonyms, because I claim that there is no real difference between them. Philosophers build the semantic differences and they only have a conceptual value, not an ontological, or real, one.

5 In this sense, it is interesting to remark on the attempts to finally hide the double ontology underlying the scientific analysis of consciousness conducted by scholars like Panksepp, (Panksepp and Biven, 2012). The neuroscientist proposes the phasing out of the words “brain” and “mind,” because they are residues of Cartesian dualism, but he insists on using them together: *mindbrain* or *brainmind* to encourage cultural approval of a perfect physical identity between the brain and the mind, experience and neural networks, experience and representations.

6 From now on, I will call these perspectives Constructivist Theories of the Self by using the acronym CTS.

7 I call the variant of the cognitive-conceptual model, the “ecological approach.” By means of this label, I intend to bring together all the embodied and enactive approaches to experience. All these approaches highlight the complex relational aspect between the brain, the body and the environment to justify the non-physical emergence of conscious experience (O’Regan and Noë, 2001; Noë, 2004; Thompson, 2007).

8 I will call this perspective the Matricial Theory of the Self (MTS).

9 See also Kant (1787) and Rorty (1970) about experience.

10 Manzotti (2017a,b) further expands on the idea of incorrigibility of perception and discusses different cases of perceptive illusions in a Neo Naturalist way.

11 One question can be raised: is it still necessary to use the concept of Self? What is the difference between the Self and the mind? We are aware of this problem, but it is beyond the scope of this paper.

12 From now I will refer to the Rubber Hand Illusion paradigm using the acronyms RHI.

13 See Manzotti (2009) for a better understanding of the issues at hand.

## References

[B1] Armel K. C. Ramachandran V. S. (2003). Projecting sensations to external objects: evidence from skin conductance response. *Proc. R. Soc. Lond. Ser. B* 270 1499–1506. 10.1098/rspb.2003.2364 12965016 PMC1691405

[B2] Armstrong D. M. (1968). *A Materialist Theory of the Mind.* London: Routledge and Kegan Paul.

[B3] Austin J. L. (1962). *Sense and Sensibilia.* Oxford: Oxford University Press.

[B4] Ayer A. J. (1940). *The Foundations of Empirical Knowledge.* London: MacMillan.

[B5] Bergson H. (1896). *Matter and Memory.* New York, NY: Zone Books 1994.

[B6] Botvinick M. Cohen J. (1998). Rubber hands “feel” touch that eyes see. *Nature* 391:756. 10.1038/35784 9486643

[B7] Brewer B. (1995). “Bodily awareness and the self,” in *The Body and the Self* eds Bermudez J. L. Marcel A. J. Eilan N. M. (Cambridge, MA: MIT Press) 291–303.

[B8] Bruner J. (1987). *Actual Minds, Possible Worlds.* Cambridge, MA: Harvard University Press.

[B9] Byrne A. Logue H. (2009). *Disjunctivism. Contemporary Readings.* Cambridge, MA: The MIT Press.

[B10] Cassam Q. (1997). *Self and World.* Oxford: Clarendon Press.

[B11] Cassam Q. (ed.). (1994). *Self Knowledge.* Oxford: Oxford University Press.

[B12] Chalmers D. J. (1996). *The Conscious Mind: In Search of a Fundamental Theory.* New York, NY: Oxford University Press.

[B13] Churchland P. S. (2013). *Touching a Nerve. The Self as Brain.* New York, NY: W.W. Norton Company.

[B14] Crick F. (1994). *The Astonishing Hypothesis: The Scientific Search for the Soul.* New York, NY: Touchstone.

[B15] Damasio A. (1999). *The Feeling of What Happens.* New York, NY: Harcourt.

[B16] Damasio A. (2010). *Self Comes to Mind: Constructing the Conscious Brain.* New York, NY: Pantheon Books.

[B17] De Vignemont F. (2010). Embodiment, ownership and disownership. *Conscious. Cogn.* 20 82–93. 10.1016/j.concog.2010.09.004 20943417

[B18] Dehaene S. (2014). *Consciousness and the Brain: Deciphering how the Brain Codes our Thoughts.* London: Penguin Books.

[B19] Dehaene S. Changeux J.-P. P. (2011). Experimental and theoretical approaches to conscious processing. *Neuron* 70 200–227. 10.1016/j.neuron.2011.03.018 21521609

[B20] Dennett D. C. (1988). Why everyone is a novelist? *Times Lit. Suppl.* 1016 1028–1029.

[B21] Dennett D. C. (1992). “The self as the centre of narrative gravity,” in *Self and Consciousness: Multiple Perspectives* eds Kessel F. S. Cole P. M. Johnson D. L. (Hillsdale, NJ: Erlbaum) 103–115.

[B22] Dennett D. C. (1996). *Kinds of Minds: Toward an Understanding of Consciousness*, 1st Edn, Vol. 4 New York, NY: Basic Books

[B23] Descartes R. (1637). *Discourse on the Method.* Indianapolis: Hackett Publishing Company.

[B24] Descartes R. (1641). *Meditations, in the Philosophical Writings of Descartes* Vol. 2 trans. Cottingham J. Stoothof R. Murdoch D. Kenny A. (Cambridge: Cambridge University Press) 1985.

[B25] Devinsky O. (2000). Right cerebral hemisphere dominance for a sense of corporeal and emotional self. *Epilepsy Behav.* 1 60–73. 10.1006/ebeh.2000.0025

[B26] Einstein A. (1916). *Relativity.* London: Routledge 10.4324/9780203198711

[B27] Engel A. K. Singer W. (2001). Temporal binding and the neural correlates of awareness. *Trends Cogn. Sci.* 5 16–25. 10.1016/S1364-6613(00)01568-0 11164732

[B28] Farrer C. Frith C. (2002). Experiencing oneself vs. another person as being the cause of an action: the neural correlates of the experience of agency. *Neuroimage* 15 596–603. 10.1006/nimg.2001.1009 11848702

[B29] Galilei G. (1623). *The Assayer. In Discoveries and opinions of Galileo* trans. Drake S. (Garden City, NY: Masterworks Program).

[B30] Gallagher S. (2000). Philosophical conceptions of the self: implications for cognitive science. *Trends Cogn. Sci.* 4 14–21. 10.1016/S1364-6613(99)01417-5 10637618

[B31] Gallagher S. (2017). *Enactivist Interventions. Rethinking the Mind.* Oxford: Oxford University Press 10.1093/oso/9780198794325.001.0001

[B32] Gallese V. Ferri F. (2014). Psychopathology and the bodily self. The case of schizophrenia. *Psychopathology* 47 357–364. 10.1159/000365638 25359279

[B33] Gallese V. Sinigaglia C. (2010). The bodily self as power for action. *Neuropsychologia* 48 746–755. 10.1016/j.neuropsychologia.2009.09.038 19835895

[B34] Gallese V. Sinigaglia C. (2011). How the body in action shapes the self. *J. Conscious. Stud.* 18 117–143.

[B35] Gergen K. (2011). “The social construction of the self,” in *The Oxford Handbook of the Self* ed. Gallagher S. (Oxford: Oxford University Press) 633–653.

[B36] Gilbert S. F. Tauber A. I. (2016). Rethinking individuality: the dialectics of the holobiont. *Biol. Philos.* 31 839–853. 10.1007/s10539-016-9541-3

[B37] Hinton J. M. (1973). *Experiences.* Oxford: Clarendon Press.

[B38] Hume D. (1758). *An Enquiry Concerning Human Understanding.* Chicago, IL: Gateway.

[B39] Husserl E. (1923). *Erste Philosophie, Parts I and II, Husserliana VII and VIII* ed. Boehm R. (Den Haag: Nijhoff).

[B40] Husserl E. (1954). *The Crisis of European Sciences.* Evanston, IL: Northwestern University Press 1970.

[B41] Husserl E. (1901/1902). *Logical Investigations* trans. Findlay J. N. (London: Routledge) 1973.

[B42] Kant I. (1787). *The Critique of Pure Reason* trans. Smith N. K. (London: Macmillan) 1933.

[B43] Kim J. (2005). *Physicalism or Something Near Enough.* Princeton, NJ: Princeton University Press.

[B44] Kircher T. David A. (2003). *The Self in Neuroscience and Psychiatry.* Cambridge: Cambridge University Press 10.1017/CBO9780511543708

[B45] Kircher T. T. Senior C. Phillips M. L. Benson P. J. Bullmore E. T. Brammer M. (2000). Towards a functional neuroanatomy of self-processing: effects of faces and words. *Cogn. Brain Res.* 10 133–144. 10.1016/S0926-6410(00)00036-7 10978701

[B46] Koch C. (2004). *The Quest for Consciousness: A Neurobiological Approach.* Englewood, NJ: Roberts & Company Publishers.

[B47] Lewis D. K. (1986). *Philosophical Papers* Vol. II. Oxford: Oxford University Press.

[B48] Locke J. (1694). *An Essay Concerning Human Understanding* ed. Nidditch P. (Oxford: Clarendon Press).

[B49] Manzotti R. (2009). No times, no wholes. A temporal and causal-oriented approach to the ontology of wholes. *Axiomathes* 19 193–214. 10.1007/s10516-009-9067-2

[B50] Manzotti R. (2017a). *Consciousness and Object. A Mind-Object Identity Physicalist theory.* Amsterdam: John Benjamins 10.1075/aicr.95

[B51] Manzotti R. (2017b). *The Spread Mind.* New York, NY: OR books.

[B52] Marr D. (1982). *Vision.* San Francisco, CA: Freeman.

[B53] McDowell J. (1982). Criteria, defeasibility and knowledge. *Proc. Br. Acad.* 68 455–479.

[B54] McKay M. D. (1978). Selves and brains. *Neuroscience* 3 599–606. 10.1016/0306-4522(78)90001-5 724109

[B55] McKay M. D. (1980). The interdependence of mind and brain. *Neuroscience* 5 1389–1391. 10.1016/0306-4522(80)90211-0 7402478

[B56] Melzack R. Israel R. Lacroix R. Schultz G. (1997). Phantom limbs in people with congenital limb deficiency or amputation in early childhood. *Brain* 120 1603–1620. 10.1093/brain/120.9.1603 9313643

[B57] Merricks T. (2001). *Objects and Persons.* Oxford: Clarendon Press 10.1093/0199245363.001.0001

[B58] Metzinger T. (2003). *Being no One.* Cambridge, MA: MIT Press 10.7551/mitpress/1551.001.0001

[B59] Miller G. A. (1983). “Informavores,” in *The Study of Information: Interdisciplinary Messages* ed. Machlup Una F. M. (New York, NY: Wiley-Interscience) 111–113.

[B60] Nagel T. (1974). What is it like to be a bat? *Philos. Rev.* 4 435–450. 10.2307/2183914

[B61] Noë A. (2004). *Action in Perception.* Cambridge, MA: MIT Press.

[B62] Northoff G. Heinzel A. de Greck M. Bermpohl F. Dobrowolny H. Panksepp J. (2006). Self-referential processing in our brain–a meta-analysis of imaging studies on the self. *Neuroimage* 31 440–457. 10.1016/j.neuroimage.2005.12.002 16466680

[B63] Nozick R. (1981). *Philosophical Explanations.* Cambridge, MA: Harvard University Press.

[B64] O’Regan J. K. (2011). *Why Red Doesn’t Sound Like a Bell: Understanding the Feel of Consciousness.* New York, NY: Oxford University Press 10.1093/acprof:oso/9780199775224.001.0001

[B65] O’Regan J. K. Noë A. (2001). A sensorimotor account of vision and visual consciousness. *Behav. Brain Sci.* 24 883–917. 10.1017/S0140525X01000115 12239892

[B66] Panksepp J. Biven L. (2012). *The Archeology of the Mind.* New York, NY: W.W. Norton & Company.

[B67] Perry J. (1975). *Personal Identity.* Berkeley, CA: University of California Press.

[B68] Perry J. (2001). *Knowledge. Possibility and Consciousness.* Cambridge, MA: MIT Press 10.7551/mitpress/4077.001.0001

[B69] Perry J. (2002). *Identity. Personal Identity and the Self.* Indianapolis: Hackett Publishing.

[B70] Pinker S. (1997). *How the Mind Works.* New York, NY: W. W. Norton. 10.1111/j.1749-6632.1999.tb08538.x 10415890

[B71] Platek S. M. Critton S. R. Myers T. E. Gallup G. G. Jr. (2003). Contagious yawning: the role of self-awareness and mental state attribution. *Cogn. Brain Res.* 17 223–227. 10.1016/S0926-6410(03)00109-5 12880893

[B72] Pradeau T. (2016). Organisms or biological individuals? Combining physiological and evolutionary individuality. *Biol. Philos.* 31 797–817. 10.1007/s10539-016-9551-1

[B73] Pradeau T. Carosella E. (2006). The self model and the conception of biological identity in immunology. *Biol. Philos.* 21 235–252. 10.1007/s10539-005-8621-6 16197907

[B74] Rorty R. (1970). Incorrigibility a S the mark of the mental. *J. Philos.* 68 399–424. 10.2307/2024002

[B75] Rovelli C. (2017). *L’ordine del Tempo.* Milano: Adelphi.

[B76] Russell B. (1912). *The Problems of Philosophy.* London: T. Butterworth.

[B77] Searle J. R. (1984). *Minds, Brains, and Science.* Cambridge, MA: Harvard University Press.

[B78] Sellars W. (1962). “Philosophy and the scientific image of man,” in *Frontiers of Science and Philosophy* ed. Colodny R. (Pittsburgh, PA: University of Pittsburgh Press) 35–78.

[B79] Shoemaker S. (1968). Self-reference and self-awareness. *J. Philos.* 65 556–579. 10.2307/2024121

[B80] Shoemaker S. (1970). Persons and their pasts. *Am. Philos. Q.* 7 269–285.

[B81] Shoemaker S. (1994). Phenomenal character. *Noûs* 28 21–38. 10.2307/2215918

[B82] Sider T. (2001). *Four Dimensionalism. An Ontology of Persistence and Time.* Oxford: Oxford Clarendon Press 10.1093/019924443X.001.0001

[B83] Simons P. M. (1987). *Parts. A Study in Ontology.* Oxford: Clarendon Press.

[B84] Smart J. J. C. (1959). “Sensations and brain processes,” in *The Philosophical Review* Vol. 68 ed. Chappell V. C. (Englewood-Cliff, NJ: Prentice Hall) 141–156. 10.2307/2182164

[B85] Strawson G. (2008). *Real Materialism and other Essays.* Oxford: Oxford Clarendon Press 10.1093/acprof:oso/9780199267422.001.0001

[B86] Strawson G. (2017). *The Subject of Experience.* Oxford: Oxford University Press 10.1093/acprof:oso/9780198777885.001.0001

[B87] Strawson P. F. (1959). *Individuals.* New York, NY: Routledge.

[B88] Tauber A. I. (2013). Immunology’s theories of cognition. *His. Philos. Life Sci.* 35 239–264. 24466634

[B89] Thompson E. (2007). *Mind in Life: Biology, Phenomenology, and the Sciences of Mind.* Cambridge, MA: Harvard University Press.

[B90] Thompson E. (2015). *Waking, Dreaming, Being: Self and Consciousness in Neuroscience, Meditation, and Philosophy.* New York, NY: Columbia University Press.

[B91] Tononi G. (2004). An information integration theory of consciousness. *BMC Neurosci.* 5:42. 10.1186/1471-2202-5-42 15522121 PMC543470

[B92] Tononi G. (2008). Consciousness as integrated information: a provisional manifesto. *Biol. Bull.* 215 216–242. 10.2307/25470707 19098144

[B93] Tononi G. Koch C. (2008). The neural correlates of consciousness: an update. *Ann. N. Y. Acad. Sci.* 1124 239–261. 10.1196/annals.1440.004 18400934

[B94] Tononi G. Koch C. (2014). Consciousness: here, there but not everywhere. *Towards Sci. Conscious.* 2014 1–20.

[B95] Tononi G. Laureys S. (2009). “The neurology of consciousness: an overview,” in *The Neurology of Consciousness*, eds Laureys S. Tononi G. (London: Academic Press), 375–412. 10.1016/B978-0-12-374168-4.00028-9

[B96] Tsakiris M. (2010). My body in the brain: a neurocognitive model of body-ownership. *Neuropsychologia* 48 703–712. 10.1016/j.neuropsychologia.2009.09.034 19819247

[B97] Tsakiris M. Haggard P. (2005). The rubber hand illusion revisited: visuotactile integration and self-attribution. *J. Exp. Psychol. Hum. Percept. Perform.* 31 80–91. 10.1037/0096-1523.31.1.80 15709864

[B98] Tsakiris M. Hesse M. D. Boy C. Haggard P. Fink G. R. (2006). Neural signatures of body ownership: a sensory network for bodily self-consciousness. *Cereb. Cortex* 17 2235–2244. 10.1093/cercor/bhl131 17138596

[B99] Van Inwagen P. (1990). *Material Beings.* New York, NY: Cornell University Press.

[B100] Varzi A. (1996). Parts, wholes, and part-whole relations: the prospects of mereotopology. *Data Knowl. Eng.* 20 259–286. 10.1016/S0169-023X(96)00017-1

[B101] Vogeley K. Bussfeld P. Newen A. Herrmann S. Happe F. Falkai P. (2001). Mind reading: neural mechanisms of theory of mind and self-perspective. *Neuroimage* 14 170–181. 10.1006/nimg.2001.0789 11525326

[B102] Vogeley K. Fink G. R. (2003). Neural correlates of the first-person-perspective. *Trends Cogn. Sci.* 7 38–42. 10.1016/S1364-6613(02)00003-7 12517357

[B103] Vogeley K. Gallagher S. (2011). “The self in the brain,” in *The Oxford Handbook of the Self* ed. Gallagher S. (Oxford: Oxford University Press) 111–136.

[B104] Whitehead A. N. (1920). *Concept of Nature.* Cambridge, MA: Cambridge University Press.

[B105] Whitehead A. N. (1925). *Science and the Modern World.* New York, NY: Macmillan Company.

[B106] Zahavi D. (2005). *Subjectivity and Selfhood: Investigating the First-Person Perspective.* Cambridge, MA: MIT Press 10.7551/mitpress/6541.001.0001

[B107] Zahavi D. (2014). *Self and Other. Exploring Subjectivity, Empathy, and Shame.* Oxford: Oxford University Press 10.1093/acprof:oso/9780199590681.001.0001

[B108] Zeki S. Bartels A. (1999). Toward a theory of visual consciousness. *Conscious. Cogn.* 8 225–259. 10.1006/ccog.1999.0390 10448004

